# Stress during Pregnancy and Offspring Pediatric Disease: A National Cohort Study

**DOI:** 10.1289/ehp.1003253

**Published:** 2011-07-21

**Authors:** Marion Tegethoff, Naomi Greene, Jørn Olsen, Emmanuel Schaffner, Gunther Meinlschmidt

**Affiliations:** 1 Division of Clinical Psychology and Psychiatry, and; 2 Division of Clinical Psychology and Psychotherapy, Department of Psychology, University of Basel, Basel, Switzerland; 3 Department of Neurobehavioral Genetics, Institute of Psychobiology, University of Trier, Trier, Germany; 4 Department of Epidemiology, School of Public Health, University of California at Los Angeles, Los Angeles, California, USA; 5 The Danish Epidemiology Science Centre, Department of Epidemiology, Institute of Public Health, University of Aarhus, Aarhus, Denmark; 6 Division of Applied Statistics in Life Sciences, Department of Psychology, University of Basel, Basel, Switzerland; 7 National Centre of Competence in Research, Swiss Etiological Study of Adjustment and Mental Health (sesam), Basel, Switzerland; 8 Biostatistics and Computational Sciences, Department of Epidemiology and Public Health, Swiss Tropical and Public Health Institute, Basel, Switzerland; 9 Division of Clinical Psychology and Epidemiology, Department of Psychology, University of Basel, Basel, Switzerland

**Keywords:** antenatal stress, child health and development, intrauterine exposure, prenatal exposure delayed effects, prenatal programming, psychosocial stress

## Abstract

Background: Identifying risk factors for adverse health outcomes in children is important. The intrauterine environment plays a pivotal role for health and disease across life.

Objectives: We conducted a comprehensive study to determine whether common psychosocial stress during pregnancy is a risk factor for a wide spectrum of pediatric diseases in the offspring.

Methods: The study was conducted using prospective data in a population-based sample of mothers with live singleton births (*n* = 66,203; 71.4% of those eligible) from the Danish National Birth Cohort. We estimated the association between maternal stress during pregnancy (classified based on two *a priori*–defined indicators of common stress forms, life stress and emotional stress) and offspring diseases during childhood (grouped into 16 categories of diagnoses from the *International Classification of Diseases, 10th Revision*, based on data from national registries), controlling for maternal stress after pregnancy.

Results: Median age at end of follow-up was 6.2 (range, 3.6–8.9) years. Life stress (highest compared with lowest quartile) was associated with an increased risk of conditions originating in the perinatal period [odds ratio (OR) = 1.13; 95% confidence interval (CI): 1.06, 1.21] and congenital malformations (OR=1.17; CI: 1.06, 1.28) and of the first diagnosis of infection [hazard ratio (HR) = 1.28; CI: 1.17, 1.39], mental disorders (age 0–2.5 years: HR = 2.03; CI: 1.32, 3.14), and eye (age 0–4.5 years: HR = 1.27; CI: 1.06, 1.53), ear (HR = 1.36; CI: 1.23, 1.51), respiratory (HR = 1.27; CI; 1.19, 1.35), digestive (HR = 1.23; CI: 1.11, 1.37), skin (HR = 1.24; CI: 1.09, 1.43), musculoskeletal (HR = 1.15; CI: 1.01–1.30), and genitourinary diseases (HR = 1.25; CI; 1.08, 1.45). Emotional stress was associated with an increased risk for the first diagnosis of infection (HR = 1.09; CI: 1.01, 1.18) and a decreased risk for the first diagnosis of endocrine (HR = 0.81; CI; 0.67, 0.99), eye (HR = 0.84; CI; 0.71, 0.99), and circulatory diseases (age 0–3 years: HR = 0.63; CI: 0.42, 0.95).

Conclusions: Maternal life stress during pregnancy may be a common risk factor for impaired child health. The results suggest new approaches to reduce childhood diseases.

Early-life factors can predispose individuals to diseases over the life course (Gluckman et al. 2008). Follow-up studies have shown that fetal growth impairment is linked to an increased risk of morbidity and premature mortality (Barker et al. 1993; Forsen et al. 2000). However, fetal growth is probably only a crude indicator of complex developmental processes that are subject to genetic factors and various intrauterine exposures that may affect gene expression and influence disease susceptibility. Therefore, the effects of intrauterine exposures on postnatal outcomes should be studied more directly (Gillman 2002), and the National Institutes of Health and the World Health Organization (WHO) have advised that the role of maternal stress during pregnancy should be given high research priority (National Institutes of Health 2003; WHO 2006). Follow-up studies have raised concerns that offspring of mothers exposed to stress during pregnancy may have an increased risk of specific diseases such as malformations, asthma, and mental and behavioral disorders (Cookson et al. 2009; Hansen et al. 2000; O’Connor et al. 2002). However, a comprehensive study covering a larger spectrum of diseases is needed.

The purpose of this study was to assess the association between common psychosocial stress during pregnancy and the risk of a wide range of offspring diseases in a population-based birth cohort with prospective data linked with a national hospital register. The decision to give an overview of a broad range of diseases accounts for the large body of evidence linking maternal adversities with changes in numerous physiological systems in the offspring (Entringer et al. 2008; Field et al. 2002; Lin et al. 2004; Monk et al. 2004, 2011; O’Connor et al. 2005).

## Methods

*Study cohort.* This study is based on prospective data from the Danish National Birth Cohort, including births between 1996 and 2003 (Olsen et al. 2001). Participants gave written informed consent, and the Danish National Committee for Biomedical Research Ethics, Copenhagen, approved the study on behalf of all committees in the country. About 50% of all general practitioners in the country took part in the recruitment, and 60% of the invited women participated. Of these, we considered as eligible all pregnancies with live singleton births (see “Results”).

*Stress exposure.* We obtained information on maternal stress from a telephone interview taken around 30 weeks of gestation. We studied two *a priori*–defined types of stress—emotional stress and life stress—as used in previous studies (Sondergaard et al. 2003; Tegethoff et al. 2010a, 2010b). The separation of these two types of maternal stress is theoretically based on the well-described stressor specificity of biological responses, which may differentially affect the fetus (Pacak and Palkovits 2001; Richardson et al. 2006). Virtually all available studies investigating maternal stress have used one of these two types of stress measures (Beydoun and Saftlas 2008; Lobel 1994). The applied instruments were developed to be feasible in a large cohort study. Emotional stress and life stress were each assessed by nine questions, each covering the time period since the beginning of pregnancy. Answers (no = 0, a little = 1, a lot = 2) were added up into a score for each stress type (range, 0–18). The scale for emotional stress covers self-reported feelings (e.g., anxiety, nervousness; for detailed description of items, see Table 1). Items were selected from the Symptom Check List-90 (Derogatis 1977) and The General Health Questionnaire (Goldberg 1972) to cover frequent symptoms of anxiety and depression. Life stress was assessed using an inventory based on the major categories of the Life Events Questionnaire (Norbeck 1984) on whether the women felt burdened in major areas of life such as work, housing, and human relations (for detailed description of items, see Table 1). For additional information on scale validation, see Supplemental Material, Additional Methods 1 (http://dx.doi.org/10.1289/ehp.1003253). We addressed up to two missing answers per stress type by using person-specific mean substitution. Women with more than two missing answers were excluded. In the analyses, we included life stress and emotional stress as categorical independent variables, with the limits between the categories defined by cutoffs as close as possible to the quartiles of the distributions of the respective stress scores (Table 2). Even though some overlap of emotional stress and life stress was expected, a low correlation between these constructs (Spearman rho correlation: *r* = 0.46; *p* < 0.001) affirmed their discriminant validity. To account for overlap, the analysis of the associations between life stress and child diseases was adjusted for emotional stress, and vice versa.

**Table 1:** Items used to assess life stress and emotional stress.

Life stress		Emotional stress	
Have you felt burdened during pregnancy by any of the things I am going to ask now. You may answer: no, a little, or a lot.		Now I am going to ask you how you have been feeling during pregnancy. You may answer: no, a little, or a lot.	
Have you been burdened by . *. .*			Have you felt . . .	
1.	*. . . *financial circumstances?		1.	*. . . *scared for no reason?
2.	*. . . *your housing situation?		2.	*. . . *hopeless about the future?
3.	*. . . *your work situation?		3.	*. . . *constantly under strain?
4.	*. . . *the relationship to your partner?		4.	*. . . *nervous or shaky inside?
5.	*. . . *relationships to family and friends?		5.	*. . . *blue?
6.	*. . . *your pregnancy?		6.	*. . . *easily annoyed or irritated?
7.	*. . . *own diseases?		7.	*. . . *that everything was an effort?
8.	*. . . *disease of your partner, family members, or close friends?		8.	*. . . *tense or keyed up?
9.	*. . . *other things?		9.	*. . . *that everything was getting on top of you?


**Table 2:** Sample characteristics (*n *= 66,203).

Characteristic	*n* (%)*a*	
Maternal age (years)		
< 27	13,614	(20.6)
27–29	17,880	(27.0)
30–32	16,517	(25.0)
> 32	18,192	(27.5)
Parity		
Primiparous	29,574	(44.7)
Multiparous	34,240	(51.7)
Unknown	2,389	(3.6)
General maternal health		
Very good	33,454	(50.5)
Average	28,114	(42.5)
Bad	2,263	(3.4)
Unknown	2,372	(3.6)
Socioeconomic status		
High	32,627	(49.3)
Medium	23,299	(35.2)
Low	5,220	(7.9)
Unknown	5,057	(7.6)
Infant sex		
Male	33,779	(51.0)
Female	32,424	(49.0)
Hypertension during pregnancy		
Yes	8,824	(13.3)
No	57,192	(86.4)
Unknown	187	(0.3)
Gestational diabetes		
Yes	1,632	(2.5)
No	64,225	(97.0)
Unknown	346	(0.5)
Smoking		
Yes	17,020	(25.7)
No	47,255	(71.4)
Unknown	1,928	(2.9)
Maternal life stress during pregnancy*b* (score)		
Low (0)	19,793	(29.9)
Low-medium (> 0 to ≤ 1)	14,488	(21.9)
Medium-high (> 1 to ≤ 3)	20,377	(30.8)
High (> 3)	11,545	(17.4)
Maternal emotional stress during pregnancy*b* (score)		
Low (≤ 1)	25,083	(37.9)
Low-medium (> 1 to ≤ 2)	11,432	(17.3)
Medium-high (> 2 to ≤ 4)	15,971	(24.1)
High (> 4)	13,717	(20.7)
Maternal life stress after pregnancy*b* (score)		
Low (0)	32,296	(48.8)
Low-medium (> 0 to ≤ 1)	13,829	(20.9)
Medium-high (> 1 to ≤ 2)	9,725	(14.7)
High (> 2)	10,353	(15.6)
Maternal emotional stress after pregnancy*b* (score)		
Low (0)	22,313	(33.7)
Low-medium (> 0 to ≤ 1)	12,557	(19.0)
Medium-high (> 1 to ≤ 3)	15,847	(23.9)
High (> 3)	15,486	(23.4)
**a**Percentages may not total 100 because of rounding. **b**Stress groups were defined by the closest possible cutoffs to the quartiles of their distribution.****		

*Outcome measures.* Information on children’s diseases was derived from the Danish National Hospital Register (Copenhagen, Denmark), which contains information on all inpatients and outpatients in Danish hospitals and provides reporting of diagnoses, the validity of which has been demonstrated for several diseases (Andersen et al. 1999; Christensen et al. 2007; Moth et al. 2007; Sorensen and Larsen 1994; Vestergaard et al. 2006). All hospital diagnoses, based on the Danish version of the *International Classification of Diseases, 10th Revision* (ICD-10) (Danish National Board of Health 1993), were divided into major diagnostic categories (dichotomous) selected *a priori* according to chapters 1–14 and 16 and 17 of the ICD-10, which cover all relevant diagnoses during childhood. Additionally, we used a dichotomous overall category (“any disease”), indicating whether any disease in any of the categories had been diagnosed [see Supplemental Material, Table 1 (http://dx.doi.org/10.1289/ehp.1003253) for details concerning the major diagnostic categories and numbers of diagnoses for specific outcomes within each category in the study cohort].

*Statistical analyses.* We used the Wilcoxon signed-rank test to compare the score reflecting maternal stress during pregnancy with the score reflecting maternal stress after pregnancy for each stress type.

For conditions that may have onset after the perinatal period (diagnostic categories 1–14) and for “any disease,” we estimated the associations of maternal life stress and emotional stress during pregnancy with the risk of the offspring for the first diagnosis of a disease within each major diagnostic category by conducting separate Cox proportional hazards regression models for each of the diagnostic categories. Data on timing of competing events, such as deaths and emigration, were not available in this data source, but these events are uncommon in Denmark (WHO 2005). In each Cox proportional hazards regression model, data on all children without a diagnosis in the respective category were censored at the end of follow-up (31 December 2006). Age in days was used as time variable. We tested the proportional hazards assumption by visually checking Schoenfeld residuals and the test of Grambsch and Therneau. If the assumption was not met, we stratified the data by age (Table 3) based on the Schoenfeld residuals to ensure that the proportional hazards assumption was met. For conditions originating in the perinatal period and for malformations, we used separate logistic regression models to estimates associations with maternal life stress and emotional stress. Low-stress groups were used as reference categories. We tested for trends using log-rank–based trend tests of the survivor function stratifying for the other covariates or by repeating logistic regressions with the stress categories modeled as a continuous ordinal variable.

**Table 3:** Cox regression models of offspring diseases predicted by stress during pregnancy.

ICD-10 category and name	No. of children with a diagnosis	Life stress Adjusted*a* HR (95% CI)								Life stress *p* for trend	Emotional stress Adjusted*a* HR (95% CI)								Emotional stress *p* for trend
		Low-medium vs. low		Medium-high vs. low		High vs. low		Low-medium vs. low			Medium-high vs. low		High vs. low						
1. Infections, parasitic diseases		6,674		1.08	(1.00, 1.16)		1.10	(1.03, 1.18)		1.28	(1.17, 1.39)		< 0.001		0.99	(0.92, 1.07)		1.06	(0.99, 1.13)		1.09	(1.01, 1.18)		0.008
2. Neoplasms		711		0.93	(0.75, 1.16)		1.06	(0.86, 1.30)		0.94	(0.73, 1.22)		0.99		0.96	(0.76, 1.20)		1.07	(0.86, 1.32)		1.13	(0.89, 1.44)		0.35
3. Diseases of blood, immune system		512		1.01	(0.78, 1.31)		1.01	(0.78, 1.30)		1.19	(0.89, 1.59)		0.49		1.17	(0.90, 1.52)		1.17	(0.91, 1.50)		1.12	(0.85, 1.48)		0.51
4. Endocrine, metabolic disorders		1,178		1.03	(0.87, 1.22)		1.09	(0.93, 1.28)		1.20	(0.99, 1.47)		0.09		1.03	(0.87, 1.21)		0.87	(0.74, 1.03)		0.81	(0.67, 0.99)		0.02
5. Mental and behavioral disorders*b*		543													1.12	(0.87, 1.44)		0.88	(0.68, 1.13)		0.86	(0.65, 1.14)		0.076
≤ 2.5 years				1.49	(0.98, 2.25)		1.34	(0.90, 2.01)		2.03	(1.32, 3.14)		0.007											
> 2.5 years				0.78	(0.55, 1.10)		1.06	(0.78, 1.43)		1.11	(0.78, 1.58)		0.747											
6. Diseases of nervous system		1,268		1.00	(0.85, 1.18)		1.06	(0.90, 1.23)		1.17	(0.96, 1.42)		0.21		0.85	(0.72, 1.01)		0.97	(0.83, 1.13)		0.86	(0.72, 1.04)		0.15
7. Diseases of eye*b*		1,451																						
≤ 4.5 years				0.98	(0.83, 1.16)		0.99	(0.84, 1.15)		1.27	(1.06, 1.53)		0.34		0.95	(0.82, 1.11)		0.98	(0.84, 1.13)		0.84	(0.71, 0.99)		0.14
> 4.5 years				1.27	(0.89, 1.82)		1.62	(1.18, 2.22)		1.19	(0.80, 1.78)		0.13											
8. Diseases of ear		4,344		1.04	(0.95, 1.14)		1.15	(1.05, 1.25)		1.36	(1.23, 1.51)		< 0.001		1.06	(0.97, 1.16)		1.03	(0.95, 1.12)		1.04	(0.95, 1.15)		0.25
9. Diseases of circulatory system*b*		362		1.14	(0.85, 1.54)		1.03	(0.77, 1.38)		1.29	(0.91, 1.84)		0.07											
≤ 3.0 years															0.84	(0.57, 1.24)		0.70	(0.49, 1.02)		0.63	(0.42, 0.95)		0.08
> 3.0 years															0.95	(0.58, 1.55)		0.61	(0.37, 1.01)		1.16	(0.74, 1.82)		0.93
10. Diseases of respiratory system		12,442		1.04	(0.99, 1.10)		1.14	(1.08, 1.20)		1.27	(1.19, 1.35)		< 0.001		0.98	(0.93, 1.04)		0.97	(0.92, 1.02)		1.00	(0.94, 1.06)		0.98
11. Diseases of digestive system		4,032		1.06	(0.97, 1.17)		1.21	(1.11, 1.31)		1.23	(1.11, 1.37)		< 0.001		0.99	(0.90, 1.09)		1.03	(0.95, 1.13)		0.97	(0.87, 1.07)		0.71
12. Diseases of skin		2,500		0.95	(0.85, 1.07)		1.06	(0.95, 1.19)		1.24	(1.09, 1.43)		0.005		0.97	(0.86, 1.09)		0.92	(0.82, 1.03)		0.93	(0.82, 1.06)		0.33
13. Diseases of musculoskeletal system		3,107		1.03	(0.93, 1.14)		1.06	(0.96, 1.17)		1.15	(1.01, 1.30)		0.01		0.99	(0.89, 1.10)		0.91	(0.83, 1.01)		0.99	(0.88, 1.11)		0.67
14. Diseases of genitourinary system		2,243		1.13	(1.00, 1.27)		1.19	(1.06, 1.34)		1.25	(1.08, 1.45)		0.02		0.96	(0.85, 1.09)		1.09	(0.97, 1.22)		0.97	(0.85, 1.11)		0.86
Any		34,665		1.02	(0.99, 1.05)		1.11	(1.08, 1.14)		1.16	(1.12, 1.20)		< 0.001		1.00	(0.97, 1.03)		1.01	(0.98, 1.04)		1.03	(0.99, 1.06)		0.07
HRs for the offspring having an initial diagnosis within the respective diagnostic category (outcomes) according to maternal life stress and emotional stress during pregnancy (predictors) (*n* = 66,203). Information on diagnoses of the children was obtained from the database-linked Danish National Hospital Register, which contains information on all diagnoses of inpatients and outpatients in Danish hospitals. In each diagnostic category, we used the initial diagnosis.**a**Model stratified for socioeconomic status, parity, maternal age, general maternal health, and infant sex, and adjusted for postnatal life stress and postnatal emotional stress of the mother. **b**Data were split at indicated times.																								

We calculated all standard errors using the clustered sandwich estimator to correct for possible dependence between health outcomes in infants born to the same mother (*n* = 3,029). Moreover, to control for previous reproductive experiences and their possible effect on exposures (Olsen 2008), we repeated all analyses including only the first pregnancy of each woman in the cohort.

To obtain less-confounded estimates, we adjusted for potential predictors of child health, selected *a priori*, including socioeconomic status (Gissler et al. 1998) (based on occupation of the mother; see Bech et al. 2005), parity (Dockerty et al. 2001), maternal age (Hassold and Chiu 1985), self-reported general maternal health (Waters et al. 2000), and infant sex (Gissler et al. 1999), categorized as indicated in Table 2. Moreover, for explorative purposes, we repeated the analyses, controlling for self-reported hypertension during pregnancy (Fatemeh et al. 2010), gestational diabetes (Aman et al. 2011), and maternal smoking during pregnancy (Shea and Steiner 2008) as potential mediators (categorized as yes, no, or unknown). In addition, we repeated the analyses, controlling for birth weight and length of gestation (Gillman 2002) (continuous) as potential mediators. We obtained information on socioeconomic status, parity, and general maternal health from an interview around 12 weeks of gestation; on smoking, hypertension, and gestational diabetes from interviews around 12 weeks of gestation, 30 weeks of gestation, and 6 months postpartum; and on sex, maternal age at delivery, birth weight, and length of gestation from the Danish Medical Birth Registry (Copenhagen, Denmark). We extended the model by adjusting for postnatal exposure to maternal stress assessed, according to the method described for maternal stress during pregnancy, at 6 months postpartum, covering the time since parturition. High maternal stress may be a consequence rather than a cause of offspring disease within the first 6 months of life (Fowlie and McHaffie 2004). Therefore, we repeated the analyses after excluding all cases having their first diagnosis within the first 7 months of life.

All hypothesis tests were two-tailed, with the level of significance set at 0.05. We addressed loss to follow-up and missing data by restricting analyses to mother–child pairs with complete data on stress. For those with missing information on a particular covariate, we included an additional “missing” category for the respective variable.

## Results

*Study cohort characteristics.* Complete information on maternal stress during and after pregnancy and on diagnoses was available for 66,203 (99%) of the eligible mother–child pairs that participated in all of the relevant interviews [see Supplemental Material, Figure 1 (http://dx.doi.org/10.1289/ehp.1003253) for details regarding study recruitment and observations included in this analysis]. Sample characteristics are provided in Table 2 (for additional information on the sample characteristics according to the stress categories, see Supplemental Material, Table 2). Median age at the end of follow-up was 6.2 (range, 3.6–8.9) years. Life and emotional stress were reported as stronger before than after birth (life stress: z = 94.81, *p* < 0.001; emotional stress: z = 66.18, *p* < 0.001, Wilcoxon signed-rank tests). Cumulative lifetime incidences of all diagnostic categories are shown according to quartile of prenatal life or emotional stress in Supplemental Material, Figure 2.

*Regression analyses.* After adjustment, maternal life stress during pregnancy was associated with an increased disease risk in 11 of 16 diagnostic categories, including an increased risk for the first diagnosis of infectious and parasitic diseases, mental and behavioral disorders (up to 2.5 years of age), diseases of the eye, ear, respiratory system, digestive system, skin, musculoskeletal and genitourinary systems, and of any disease (Table 3). Maternal life stress during pregnancy was associated with an increased risk of conditions originating in the perinatal period and of congenital malformations (Table 4).

**Table 4:** Logistic regression models of offspring diseases predicted by stress during pregnancy.

No. of children with a diagnosis	Life stress Adjusted*a* OR (95% CI)								Emotional stress Adjusted*a* OR (95% CI)								Emotional stress *p* for trend
	ICD-10 category and name	Low-medium vs. low		Medium-high vs. low		High vs. low		Life stress *p* for trend	Low-medium vs. low		Medium-high vs. low		High vs. low				
16. Conditions originating in perinatal period		12,590		1.03	(0.97, 1.09)		1.11	(1.06, 1.18)		1.13	(1.06, 1.21)		< 0.001		1.01	(0.95, 1.07)		1.02	(0.97, 1.07)		1.04	(0.98, 1.10)		0.24
17. Malformations		5,534		1.03	(0.95, 1.12)		1.13	(1.05, 1.21)		1.17	(1.06, 1.28)		< 0.001		1.00	(0.92, 1.08)		1.00	(0.93, 1.08)		1.03	(0.95, 1.12)		0.54
Abbreviations: CI, confidence interval; OR, odds ratio. ORs for the offspring having conditions originating in the perinatal period and malformations (outcomes) according to maternal life stress and emotional stress during pregnancy (predictors) (*n* = 66,203). **a**Models adjusted for socioeconomic status, parity, maternal age, general maternal health, and infant sex.																								

Maternal emotional stress during pregnancy was associated with an increased risk for the first diagnosis of infectious and parasitic diseases and a decreased risk for the first diagnosis of endocrine and metabolic disorders, diseases of the eye, and the circulatory system (up to 3 years of age). However, a significantly reduced risk of diseases of the eye and the circulatory system was seen only in offspring of highly stressed mothers. Crude estimates of the associations between maternal stress during pregnancy and offspring diseases (i.e., estimates not adjusted for any potential confounders) are provided in Supplemental Material, Table 3 (http://dx.doi.org/10.1289/ehp.1003253).

When we repeated the adjusted analyses using only the first pregnancy of each woman in the cohort and when we repeated the adjusted analyses controlling for smoking, hypertension, and gestational diabetes, or for birth weight and length of gestation, the estimates were of similar magnitude as those presented in Tables 3 and 4 (data not shown). Repeating the analyses after excluding cases having their first diagnosis before assessment of maternal stress after pregnancy did not appreciably change the magnitude of the significant estimates in the models [all changes in hazard ratio (HR) were < 0.10]. However, the prenatal life stress–associated risk for the first onset of mental disorders within the first 2.5 years of life decreased markedly [4th quartile vs. 1st quartile: HR =1.43; 95% confidence interval (CI): 0.82, 2.47 compared with HR = 2.03; CI: 1.32, 3.14].

## Discussion and Conclusions

In this large population-based cohort study, maternal life stress during pregnancy was associated with an increased risk of a wide range of diseases during childhood. These findings are in line with data from animal models indicating changes in different physiological systems after maternal stress during pregnancy (Fowden et al. 2006). To our knowledge, this is the first comprehensive study of the relationship between maternal stress during pregnancy and a wide spectrum of offspring diseases during childhood.

Our study has important strengths, including prospective data collection for 66,203 mother–child pairs and linkage to a comprehensive medical registry with complete information on hospital discharge diagnoses. Although associations were of low to moderate strength, our results have broad relevance for the general population, because our definition of maternal stress focuses on everyday occurrences (rather than rare disasters or severe life events), and we focused on the whole range of illnesses, including very common diseases, which suggests that a substantial part of the population may be adversely affected by maternal stress. We adjusted for several potential confounders, but our results still may be biased by residual or uncontrolled confounding by factors such as chemical exposures. Temporal sequence, consistency of findings across a variety of categories, and evidence of a dose–response relationship support the possibility of a causal link (Grimes and Schulz 2002). We controlled for potential bias by maternal stress after pregnancy, which might increase the likelihood that a mother would seek to have her child hospitalized. In addition, it is unlikely that maternal stress would influence hospital treatment, because in the Danish health care system, 99% of the population must be referred for elective hospital treatment by a general practitioner, and both referrals and hospital treatment are free of charge for patients (Strandberg-Larsen et al. 2007). Moreover, we controlled for potential bias by reverse causation between child disease and maternal stress after pregnancy. One limitation is that we did not have data on the timing of the maternal stress exposure during pregnancy, which may play a role in the relationship between stress and long-term health, given that each organ system has a specific critical period in which it is most susceptible to intrauterine perturbations (Hansen et al. 2000; Khashan et al. 2008). However, life stress and emotional stress generally reflect chronic states of adversity (McEwen and Stellar 1993). Of all eligible mother–child pairs, 72% participated in the relevant interviews, and 99% of these were included in our analyses. Given the low loss to follow-up, the high percentage of complete data, and linkage to the Danish National Hospital Register, we think measurable selection bias is unlikely. However, missing stress interview data for 7,487 mother–child pairs [8% of those eligible; Supplemental Material, Figure 1 (http://dx.doi.org/10.1289/ehp.1003253)] may have been a consequence of premature birth in some cases, as the interview was not conducted if birth had already occurred. Therefore, the observed associations between stress during pregnancy and offspring health should not be generalized to children born extremely preterm (< 30 weeks of gestation). Finally, the different ICD-10 categories are heterogeneous with regard to grouping criteria (e.g., by organ system vs. etiology), but the ICD-10 has high reliability at the category level (Stausberg et al. 2008) and is the international standard diagnostic classification system of diseases.

Our findings on maternal life stress during pregnancy corroborate and extend the results of previous studies (Hansen et al. 2000; Li et al. 2008) to common forms of maternal stress and a broader range of disease outcomes. For example, in line with other studies (Beversdorf et al. 2005; Laplante et al. 2004), our data provide evidence for an increased risk of mental disorders during the first 2.5 years of life in offspring of mothers reporting high life stress during pregnancy compared with mothers reporting low life stress. However, we cannot rule out the possibility that this result was biased by reverse causation between offspring disease and postnatal life stress. In the present study, emotional stress during pregnancy was associated with an increased risk of infectious diseases only, whereas previous studies observed associations between emotional problems during pregnancy and other outcomes, including malformations, asthma, and mental disorders (Cookson et al. 2009; O’Connor et al. 2002; Schneid-Kofman et al. 2008; Van den Bergh and Marcoen 2004; Van den Bergh et al. 2005a, 2005b). Discrepancies among studies may partly reflect differences in disease outcome or emotional stress classifications. For example, we classified outcomes based on clinical diagnoses (instead of subclinical dysfunction) in the offspring, and defined emotional stress across the entire range rather than focusing on more severe psychopathology, as in Schneid-Kofman et al. (2008). Moreover, discrepancies between findings may be explained by differential mutual control for life stress and emotional stress and by differential control for maternal stress after pregnancy.

The decreased risk of certain diseases predicted by maternal emotional stress during pregnancy is in line with some previous evidence on beneficial effects of maternal stress on offspring development and brain maturation (DiPietro et al. 2006). However, some of these associations were seen only in offspring of highly stressed mothers, with marginal statistical significance. Hence, it is too early to conclude whether common forms of maternal emotional stress during pregnancy have the potential to protect certain organ systems against disease.

The observed association between maternal stress during pregnancy and child health may represent long-term consequences of subtle adaptations in multiple organ systems to the intrauterine environment (Bateson et al. 2004). The potential biological mechanisms underlying such developmental plasticity, including epigenetic processes (Gluckman et al. 2009) and changes at the molecular, cellular, and organ level in the offspring, provide new ideas to the fetal origin of chronic disease concept (Fowden et al. 2006; Gluckman et al. 2008; Tegethoff et al. 2009). Specifically, in relation to maternal stress during pregnancy, numerous subclinical alterations in physiology, including changes in immune, brain, cardiovascular, autonomic, endocrine, and metabolic function, have been described, such as changes in fetal heart rate, insulin resistance, increased concentrations of immunoglobulin E in cord blood and changes in hypothalamic–pituitary–adrenal (HPA) axis function (Entringer et al. 2008; Field et al. 2002; Lin et al. 2004; Monk et al. 2004, 2011; O’Connor et al. 2005). Indeed, changes in HPA axis activity have been associated not only with maternal stress during pregnancy (Kapoor et al. 2008), but also with a wide range of diseases, including mental disorders (Goodyer et al. 2001), respiratory diseases (Priftis et al. 2009), diseases of the skin (Buske-Kirschbaum et al. 2010), and infectious diseases (Bailey et al. 2003). However, although the HPA axis has long been proposed as a causal link between early adversity and lifelong disease risk (Phillips 2007), it also has been suggested that HPA-related hormones may be noncausal markers of other causal mechanisms (Kramer et al. 2009, 2010). There also is some evidence that maternal stress affects placental HSD11B2, which in turn regulates the bioavailability of glucocorticoids in fetal organs (Harris and Seckl 2011). Elevated stress levels across pregnancy have also been associated with changes in production of pro-inflammatory cytokines in the offspring (Coussons-Read et al. 2007). Dysregulation of cytokine production has been associated with certain mental disorders (Conti and Fulcheri 2010; Raison et al. 2010); infectious diseases (Subauste et al. 1995); diseases of the eye, such as conjunctivitis (Niederkorn 2008); ear, such as otitis media (Smirnova et al. 2002); respiratory system, such as asthma (Finkelman et al. 2010); digestive system, such as disorders related to gastrointestinal motility (De Winter and De Man 2010); urogenital system, such as urinary tract infection (Mak and Kuo 2006); and skin, such as atopic dermatitis (Miraglia del Giudice et al. 2006). Other mechanisms, such as catecholamines (Harris and Seckl 2011), intrauterine artery resistance, and intrauterine perception and learning (Kinsella and Monk 2009), also may be involved in offspring disease programming.

As yet, we cannot exclude the possibility that associations between stress during pregnancy and child diseases are mediated by maternal behavioral factors, such as stress-related changes in lifestyle, or health-related factors. Adjusting for maternal smoking during pregnancy, hypertension, and diabetes did not alter associations between maternal stress during pregnancy and child health. Maternal nutrition is another candidate mediator, because stress is related to quality of nutrition (Torres and Nowson 2007), and a relationship between nutrition during pregnancy and a range of offspring diseases has been well documented (Symonds et al. 2007). Moreover, alcohol consumption during pregnancy has been associated with birth defects (O’Leary et al. 2010) and impaired offspring development and behavior (Bay and Kesmodel 2010; Sood et al. 2001), indicating that alcohol may mediate associations between maternal stress and child health. Adjusting for birth weight and length of gestation had little effect on associations.

The observed associations between maternal stress during pregnancy and offspring health may have implications for public health and health care policy: First, further investment in the reduction of life stress during pregnancy may be an important opportunity to improve child health. Second, our findings encourage consideration of preventive strategies for infants of mothers who were highly stressed during pregnancy.

This study suggests that maternal life stress during pregnancy may be a common risk factor for a wide range of diseases in the offspring; however, we found almost no evidence for adverse health consequences of maternal emotional stress during pregnancy. Future studies should *a*) focus on the underlying mechanisms of these relationships, *b*) analyze the associations between selected forms of maternal stress during pregnancy and specific offspring diseases, including more detailed information on disease characteristics and information from further health registries, and *c*) identify modifiable determinants within this context to improve preventive approaches and interventions.

## Supplemental Material

(892 KB) PDF
