# Supplementary material for: Stress during Pregnancy and Offspring Pediatric Disease: A National Cohort Study
Source: Environ Health Perspect. 2011 Jul 21;119(11):1647–52. doi: 10.1289/ehp.1003253 (PMC3226491; doi:10.1289/ehp.1003253)
Supplement: (892 KB) PDF [file ehp.1003253.s001.pdf]

Supplemental Material

Stress during pregnancy and offspring pediatric disease:

A national cohort study

(Tegethoff, Greene, Olsen, Schaffner, Meinlschmidt)

Table of Contents:

Supplemental Material Additional Methods 1: Information on scale validation……... 2

Supplemental Material Table 1: Descriptives of diagnostic categories in study cohort (N = 66203)……..…………………………………………………………………... 3

Supplemental Material Table 2: Sample characteristics according to stress during pregnancy..…………………………………………………………………………..… 6

Supplemental Material Table 3: Unadjusted Regression Models of Offspring Diseases Predicted by Stress During Pregnancy……………………………………… 8

Supplemental Material Figure 1: Flowchart of study participants…………………….10

Supplemental Material Figure 2: Cumulative Lifetime Incidences of Offspring Disease by Stress During Pregnancy……………………………………………………11

Supplemental Material References………………………………………………………15

Supplemental Material Additional Methods 1: Information on scale validation

To prevent somatic confounding due to physical conditions during pregnancy, we only included items related to emotional symptoms, but none related to somatic symptoms. We validated the scale according to several types of validity (Bland and Altman 2002). Internal consistency among items was satisfactory (Cronbach's alpha = 0.75). Construct validity was determined using two external validations, demonstrating that the emotional stress scale clearly distinguished between pregnant women (i) suffering versus not suffering from a mental disorder during pregnancy (two-sample t test with equal variances: $t_{1,63385} = –45.64$; $P < 0.001$; mean emotional stress score (95% confidence interval) in sufferers and non-sufferers: 6.97 (6.68–7.26) and 2.72 (2.70–2.74), respectively), and (ii) having versus not having consulted a psychologist/psychiatrist in the past ($t_{1,63799} = –53.08$; $P < 0.001$; mean emotional stress score (95% CI) in consulters and non-consulters: 4.73 (4.63–4.83) and 2.64 (2.62–2.66), respectively). Moreover, we tested the scale's relationship with the Symptom Check List-90 in a separate sample of pregnant women ($N = 64$), relating the selected items with the Symptom Check List-90 global severity index (Spearman-Rho correlation: $r = 0.917$; $P < 0.001$). We examined construct validity by testing the inventory's relationship with a standard measure of daily hassles (Kanner et al. 1981) in a separate sample of pregnant women ($N = 65$), relating nine comparable items with the total score (Spearman-Rho correlation: $r = 0.899$; $P < 0.001$).

Supplemental Material Table 1. Descriptives of Diagnostic Categories in Study Cohort (N = 66203).

ICD-10 category No. *(ICD-10 codes)*	Title	No. of children with a diagnosis	Age (in years) at initial diagnosis [median (range)]	Most frequent initial diagnoses[a] within diagnostic category
1 *(A00-B99)*	Certain infectious and parasitic diseases	6674	1.3 (0-8.3)	Viral infection, unspecified (*N* = 2711)<br>Diarrhoea and gastroenteritis of presumed infectious origin (*N* = 1709)<br>Viral intestinal infection unspecified (*N* = 726)<br>Varicella without complication (*N* = 148)<br>Septicaemia, unspecified (*N* = 80)
2 *(C00-D48)*	Neoplasms	711	1.6 (0-8.2)	Haemangioma, any site (*N* = 211)<br>Benign neoplasms of skin, unspecified (*N* = 28)<br>Benign neoplasms of skin (skin of eyelid, including canthus) (*N* = 23)<br>Benign neoplasms of connective and other soft tissue of head, face and neck (*N* = 21)<br>Acute lymphoblastic leukaemia (*N* = 19)
3 *(D50-D89)*	Diseases of the blood and blood-forming organs and certain disorders involving the immune mechanisms	512	2.3 (0-8.1)	Allergic purpura (*N* = 149)<br>Idiopathic thrombocytopenic purpura (*N* = 55)<br>Anaemia, unspecified (*N* = 45)<br>Other specified immunodeficiencies (*N* = 22)<br>Immunodeficiency, unspecified (*N* = 20)
4 *(E00-E90)*	Endocrine, nutritional and metabolic disorders	1178	1.7 (0-8.3)	Volume depletion (*N* = 559)<br>Acidosis (*N* = 141)<br>Hypoglycaemia, unspecified (*N* = 50)<br>Short stature, not elsewhere classified (*N* = 45)<br>Insulin-dependent diabetes mellitus, without complications (*N* = 40)
5 *(F00-F99)*	Mental and behavioral disorders	543	3.7 (0-8.2)	< 2.5 years<br>Feeding disorder of infancy and childhood (N = 54)<br>Eating disorder, unspecified (N = 21)<br>Unspecified mental retardation, without mention of impairment of behaviour (N = 20)<br>Adjustment disorders (N = 17)<br>Other eating disorders (N = 13)<br>> 2.5 years<br>Nonorganic encopresis (N = 51)<br>Mixed specific developmental disorders (N = 24)<br>Disturbance of activity and attention (N = 19)<br>Nonorganic enuresis (N = 18)<br>Childhood autism (N = 15)

Supplemental Material Table 1. Descriptives of Diagnostic Categories in Study Cohort (N = 66203) (cont).

6<br>*(G00-G99)*	Diseases of the nervous system	1268	2.2 (0-8.3)	Epilepsy, unspecified (*N* = 350)<br>Sleep apnoea (*N* = 157)<br>Hydrocephalus, unspecified (*N* = 45)<br>Cerebral palsy, unspecified (*N* = 44)<br>Bacterial meningitis, unspecified (*N* = 39)
7<br>*(H00-H59)*	Diseases of the eye and adnexa	1451	2.2 (0-8.2)	**< 4.5 years**<br>Acute conjunctivitis, unspecified (*N* = 219)<br>Stenosis and insufficiency of lacrimal passages (*N* = 121)<br>Convergent concomitant strabismus (*N* = 90)<br>Hypermetropia (*N* = 68)<br>Chalazion (*N* = 54)<br>**> 4.5 years**<br>Hypermetropia (*N* = 27)<br>Convergent concomitant strabismus (*N* = 26)<br>Acute conjunctivitis, unspecified (*N* = 22)<br>Chalazion (*N* = 19)<br>Acute atopic conjunctivitis (*N* = 16)
8<br>*(H60-H95)*	Diseases of the ear and mastoid process	4344	1.4 (0-8.5)	Acute suppurative otitis media (*N* = 1221)<br>Hearing loss, unspecified (*N* = 570)<br>Chronic mucoid otitis media (*N* = 515)<br>Otitis media, unspecified (*N* = 393)<br>Nonsuppurative otitis media, unspecified (*N* = 362)
9<br>*(I00-I99)*	Diseases of the circulatory system	362	2.1 (0-8.1)	**< 3 years**<br>Heart disease, unspecified (*N* = 16)<br>Nonspecific lymphadenitis, unspecified (*N* = 16)<br>Chronic lymphadenitis, except mesenteric (*N* = 15)<br>Supraventricular tachycardia (*N* = 14)<br>Atrial premature depolarization (*N* = 10)<br>**> 3 years**<br>Nonspecific lymphadenitis, unspecified (*N* = 21)<br>Chronic lymphadenitis, except mesenteric (*N* = 16)<br>Unspecified haemorrhoids without complication (*N* = 14)<br>Nonspecific mesenteric lymphadenitis (*N* = 6)<br>Supraventricular tachycardia (*N* = 6)
10<br>*(J00-J99)*	Diseases of the respiratory system	12442	1.4 (0-8.4)	Asthma, unspecified (*N* = 1873)<br>Acute obstructive laryngitis [croup] (*N* = 1574)<br>Pneumonia, unspecified (*N* = 1299)<br>Acute bronchitis, unspecified (*N* = 1152)<br>Acute nasopharyngitis [common cold] (*N* = 779)

Supplemental Material Table 1. Descriptives of Diagnostic Categories in Study Cohort (N = 66203) (cont).

11<br>*(K00-K93)*	Diseases of the digestive system	4032	2.1 (0-8.8)	Unilateral or unspecified inguinal hernia, without obstruction or gangrene (*N* = 1015)<br>Constipation (*N* = 741)<br>Umbilical hernia without obstruction or gangrene (*N* = 251)<br>Allergic and dietetic gastroenteritis and colitis (*N* = 175)<br>Gastro-oesophageal reflux disease without oesophagitis (*N* = 106)
12<br>*(L00-L99)*	Diseases of the skin and subcutaneous tissue	2500	2.1 (0-8.3)	Atopic dermatitis, unspecified (*N* = 243)<br>Besnier's prurigo (*N* = 200)<br>Staphylococcal scalded skin syndrome (*N* = 165)<br>Impetigo (*N* = 155)<br>Acute lymphadenitis of face, head and neck (*N* = 155)
13<br>*(M00-M99)*	Diseases of the musculoskeletal system and connective tissue	3107	2.6 (0-8.3)	Disorder of bone, unspecified (*N* = 356)<br>Pain in limb (*N* = 345)<br>Residual foreign body in soft tissue (*N* = 318)<br>Reactive arthropathy, unspecified (*N* = 288)<br>Torticollis (*N* = 147)
14<br>*(N00-N99)*	Diseases of the genitourinary system	2243	2.7 (0-8.3)	Urinary tract infection, site not specified (*N* = 337)<br>Redundant prepuce, phimosis and paraphimosis (*N* = 314)<br>Acute tubulo-interstitial nephritis (*N* = 274)<br>Hydrocele, unspecified (*N* = 261)<br>Acute cystitis (*N* = 175)
15[b]<br>*(O00-O99)*	Pregnancy, childbirth and the puerperium			
16<br>*(P00-P96)*	Certain conditions originating in the perinatal period	12590	Not relevant	Post-term infant, not heavy for gestational age (*N* = 1522)<br>Other heavy for gestational age infants (*N* = 1453)<br>Neonatal jaundice, unspecified (*N* = 1446)<br>Other preterm infants (*N* = 983)<br>Mild and moderate birth asphyxia (*N* = 861)
17<br>*(Q00-Q99)*	Congenital malformations, deformations and chromosomal abnormalities	5534	Not relevant	Undescended testicle, unilateral (*N* = 409)<br>Congenital malformation of heart, unspecified (*N* = 388)<br>Congenital dislocation of hip, unilateral (*N* = 295)<br>Other congenital deformities of hip (*N* = 210)<br>Undescended testicle, bilateral (*N* = 167)
„Any disease“	Any disease of the ICD-10 categories	34665	0.4 (0-8.5)	

ICD-10 = International Classification of Diseases, 10th revision (Danish National Board of Health 1993).

[a] Information on the children’s diagnoses were obtained from the database-linked Danish National Hospital Register, which contains information on all ICD-10 diagnoses of inpatients and outpatients in Dar hospitals. In each diagnostic category, we used the initial diagnosis.

[b] Not relevant for children.

Supplemental Material Table 2. Sample Characteristics According to Stress During Pregnancy.

	All mother-child pairs	Maternal life stress during pregnancy[a]				Maternal emotional stress during pregnancy[a]			
		Low	Low-medium	Medium-high	High	Low	Low-medium	Medium-high	High
Total study population (*N*)	66203	19793	14488	20377	11545	25083	11432	15971	13717
	Discrete variables: n (%[b])	*Discrete variables: %[b]*							
**Maternal age (years)**									
< 27	13614 (20.6)	20.0	19.6	20.6	22.7	17.7	20.8	21.8	24.2
27-29	17880 (27.0)	29.1	27.6	26.1	24.2	28.4	27.4	26.5	24.8
30-32	16517 (25.0)	24.9	25.7	25.2	23.7	26.0	25.1	24.7	23.2
> 32	18192 (27.5)	26.0	27.1	28.1	29.4	27.9	26.8	27.0	27.9
**Parity**									
Primiparous	29574 (44.7)	51.9	46.2	41.0	36.8	46.9	45.0	44.4	40.6
Multiparous	34240 (51.7)	44.6	50.4	55.5	58.9	49.8	51.5	51.9	55.3
Unknown	2389 (3.6)	3.4	3.4	3.5	4.3	3.3	3.4	3.7	4.2
**General maternal health**									
Very good	33454 (50.5)	60.1	52.7	46.4	38.7	58.3	51.7	46.7	39.8
Average	28114 (42.5)	35.6	41.8	46.2	48.6	36.6	42.0	46.0	49.5
Bad	2263 (3.4)	1.0	2.1	3.8	8.5	1.8	2.9	3.6	6.5
Unknown	2372 (3.6)	3.4	3.4	3.5	4.2	3.3	3.4	3.7	4.2
**Socioeconomic status**									
High	32627 (49.3)	49.6	50.5	49.9	46.3	53.6	49.6	48.1	42.6
Medium	23299 (35.2)	36.9	35.7	34.1	33.6	33.8	35.4	35.6	37.1
Low	5220 (7.9)	6.8	6.9	8.3	10.2	6.1	7.5	8.5	10.9
Unknown	5057 (7.6)	6.7	7.0	7.7	9.9	6.6	7.5	7.9	9.4
**Infant sex**									
Male	33779 (51.0)	51.8	50.8	50.5	51.0	51.7	50.6	50.7	50.5
Female	32424 (49.0)	48.2	49.3	49.5	49.1	48.3	49.4	49.3	49.5
**Hypertension during pregnancy**									
Yes	8824 (13.3)	11.4	13.1	14.0	15.8	11.6	13.2	14.1	15.8
No	57192 (86.4)	88.4	86.6	85.7	83.9	88.2	86.6	85.6	83.8
Unknown	187 (0.3)	0.2	0.3	0.3	0.4	0.2	0.3	0.3	0.4
**Gestational diabetes**									
Yes	1632 (2.5)	1.8	2.3	2.8	3.2	2.1	2.4	2.8	2.9
No	64225 (97.0)	97.9	97.2	96.6	95.9	97.6	97.0	96.7	96.3
Unknown	346 (0.5)	0.3	0.5	0.6	0.9	0.4	0.6	0.5	0.8
**Smoking**									
Yes	17020 (25.7)	21.1	23.5	27.0	34.2	19.7	23.7	28.0	35.6
No	47255 (71.4)	76.0	73.6	70.2	62.8	77.4	73.6	69.0	61.4
Unknown	1928 (2.9)	2.9	2.9	2.9	3.1	2.9	2.8	3.0	3.0
**Maternal life stress during pregnancy[a] (score)**									
Low (0)	19793 (29.9)	100.0	0.0	0.0	0.0	47.2	31.0	20.2	8.7
Low-medium (>0-≤1)	14488 (21.9)	0.0	100.0	0.0	0.0	24.6	25.1	22.7	13.4
Medium-high (>1-≤3)	20377 (30.8)	0.0	0.0	100.0	0.0	23.0	32.6	37.9	35.2
High (>3)	11545 (17.4)	0.0	0.0	0.0	100.0	5.3	11.2	19.3	42.8
**Maternal emotional stress during pregnancy[a] (score)**									
Low (≤1)	25083 (37.9)	59.8	42.5	28.4	11.4	100.0	0.0	0.0	0.0
Low-medium (>1-≤2)	11432 (17.3)	17.9	19.8	18.3	11.1	0.0	100.0	0.0	0.0
Medium-high (>2-≤4)	15971 (24.1)	16.3	25.0	29.7	26.7	0.0	0.0	100.0	0.0
High (>4)	13717 (20.7)	6.0	12.7	23.7	50.8	0.0	0.0	0.0	100.0

Supplemental Material Table 2. Sample Characteristics According to Stress During Pregnancy (cont).

**Maternal life stress after pregnancy[a] (score)**									
Low (0)	32296 (48.8)	69.0	53.3	41.2	21.8	62.8	51.4	42.9	29.1
Low-medium (>0-≤1)	13829 (20.9)	17.8	23.9	23.5	17.8	19.2	22.0	23.5	20.0
Medium-high (>1-≤2)	9725 (14.7)	8.9	13.6	17.9	20.3	11.0	14.6	16.8	19.0
High (>2)	10353 (15.6)	4.3	9.2	17.4	40.1	7.0	12.0	17.8	31.9
**Maternal emotional stress after pregnancy[a] (score)**									
Low (0)	22313 (33.7)	46.7	35.6	28.4	18.4	52.9	33.2	22.2	12.5
Low-medium (>0-≤1)	12557 (19.0)	20.6	21.1	18.6	14.2	21.5	22.4	19.0	11.4
Medium-high (>1-≤3)	15847 (23.9)	20.0	24.5	26.7	25.2	17.2	27.8	30.9	25.1
High (>3)	15486 (23.4)	12.8	18.9	26.3	42.1	8.5	16.7	27.9	51.0

[a] Stress groups were defined by cutoffs the closest possible to the quartiles of their distribution distribution.
[b] Percentages may not total 100 due to rounding.

Supplemental Material Table 3. Unadjusted Regression Models of Offspring Diseases Predicted by Stress During Pregnancy.

HRs and ORs according to unadjusted model (presented in support of transparency) for the offspring having an initial diagnosis[a] within the respective diagnostic category (outcomes) according to maternal life stress and emotional stress during pregnancy (predictors) (N = 66203).

ICD-10 Category: No. Name	No. of children with a diagnosis	Life stress: Unadjusted HR			Emotional stress: Unadjusted HR		
		Low-medium vs. Low	Medium-high vs. Low	High vs. Low	Low-medium vs. Low	Medium-high vs. Low	High vs. Low
1. Infections, parasitic diseases	6674	1.09<br>[1.02, 1.17]	1.14<br>[1.06, 1.22]	1.37<br>[1.27, 1.48]	1.02<br>[0.94, 1.09]	1.13<br>[1.05, 1.20]	1.24<br>[1.15, 1.33]
2. Neoplasms	711	0.93<br>[0.75, 1.15]	1.08<br>[0.88, 1.31]	0.97<br>[0.76, 1.25]	0.97<br>[0.77, 1.21]	1.09<br>[0.89, 1.34]	1.22<br>[0.98, 1.52]
3. Diseases of blood, immune system	512	1.02<br>[0.79, 1.32]	1.05<br>[0.82, 1.34]	1.31<br>[1.00, 1.73]	1.23<br>[0.95, 1.59]	1.23<br>[0.96, 1.57]	1.25<br>[0.96, 1.62]
4. Endocrine, metabolic disorders	1178	1.02<br>[0.86, 1.21]	1.09<br>[0.94, 1.28]	1.24<br>[1.03, 1.50]	1.07<br>[0.91, 1.27]	0.95<br>[0.81, 1.11]	0.96<br>[0.81, 1.15]
5. Mental and behavioral disorders	543	<2.5y: 1.52<br>[1.01, 2.30]<br>>2.5y: 0.80<br>[0.57, 1.13]	<2.5y: 1.38<br>[0.93, 2.05]<br>>2.5y: 1.13<br>[0.85, 1.51]	<2.5y: 2.18<br>[1.45, 3.28]<br>>2.5y: 1.39<br>[1.00, 1.93]	1.23<br>[0.96, 1.58]	1.05<br>[0.83, 1.34]	1.26<br>[0.98, 1.61]
6. Diseases of nervous system	1268	1.03<br>[0.87, 1.21]	1.10<br>[0.94, 1.28]	1.27<br>[1.06, 1.52]	0.92<br>[0.77, 1.09]	1.12<br>[0.96, 1.30]	1.12<br>[0.95, 1.32]
7. Diseases of eye[b]	1451	<4.5y: 0.98<br>[0.83, 1.16]<br>>4.5y: 1.29<br>[0.90, 1.83]	<4.5y: 1.00<br>[0.86, 1.16]<br>>4.5y: 1.62<br>[1.18, 2.21]	<4.5y: 1.32<br>[1.11, 1.58]<br>>4.5y: 1.30<br>[0.89, 1.90]	0.98<br>[0.84, 1.15]	1.06<br>[0.93, 1.22]	1.01<br>[0.86, 1.17]
8. Diseases of ear	4344	1.06<br>[0.97, 1.16]	1.18<br>[1.09, 1.28]	1.45<br>[1.32, 1.60]	1.09<br>[1.00, 1.19]	1.09<br>[1.00, 1.18]	1.17<br>[1.07, 1.27]
9. Diseases of circulatory system[b]	362	1.16<br>[0.87, 1.56]	1.06<br>[0.80, 1.41]	1.29<br>[0.93, 1.80]	<3.0y: 0.91<br>[0.62, 1.33]<br>>3.0y: 1.05<br>[0.65, 1.70]	<3.0y: 0.82<br>[0.57, 1.18]<br>>3.0y: 0.70<br>[0.43, 1.14]	<3.0y: 0.82<br>[0.55, 1.21]<br>>3.0y: 1.37<br>[0.90, 2.09]
10. Diseases of respiratory system	12442	1.06<br>[1.01, 1.12]	1.19<br>[1.13, 1.25]	1.39<br>[1.31, 1.47]	1.01<br>[0.96, 1.07]	1.03<br>[0.98, 1.08]	1.11<br>[1.05, 1.17]
11. Diseases of digestive system	4032	1.05<br>[0.96, 1.16]	1.20<br>[1.11, 1.31]	1.27<br>[1.15, 1.41]	1.03<br>[0.94, 1.13]	1.11<br>[1.02, 1.21]	1.11<br>[1.01, 1.22]
12. Diseases of skin	2500	0.97<br>[0.86, 1.09]	1.10<br>[0.99, 1.22]	1.33<br>[1.17, 1.52]	0.99<br>[0.88, 1.11]	0.96<br>[0.86, 1.07]	1.01<br>[0.90, 1.14]
13. Diseases of musculoskeletal system	3107	1.04<br>[0.93,1.15]	1.08<br>[0.98, 1.19]	1.19<br>[1.06, 1.34]	1.00<br>[0.90, 1.11]	0.94<br>[0.85, 1.04]	1.04<br>[0.94, 1.16]
14. Diseases of genitourinary system	2243	1.13<br>[1.00, 1.28]	1.21<br>[1.08, 1.36]	1.31<br>[1.14, 1.50]	0.97<br>[0.85, 1.10]	1.10<br>[0.99, 1.23]	1.02<br>[0.90, 1.15]
Any	34665	1.02<br>[0.99, 1.05]	1.12<br>[1.09, 1.15]	1.19<br>[1.15, 1.23]	1.01<br>[0.98, 1.04]	1.04<br>[1.01, 1.07]	1.09<br>[1.05, 1.12]

Supplemental Material Table 3. Unadjusted Regression Models of Offspring Diseases Predicted by Stress During Pregnancy (cont).

ICD-10 Category: No. Name	No. of children with a diagnosis	Life stress: Unadjusted OR			Emotional stress: Unadjusted OR		
		Low-medium vs. Low	Medium-high vs. Low	High vs. Low	Low-medium vs. Low	Medium-high vs. Low	High vs. Low
16. Conditions originating in perinatal period	12590	1.00 [0.95, 1.06]	1.07 [1.02, 1.13]	1.09 [1.02, 1.16]	1.01 [0.96, 1.07]	1.03 [0.98, 1.09]	1.06 [1.00, 1.12]
17. Malformations	5534	1.02 [0.94, 1.10]	1.10 [1.02, 1.18]	1.14 [1.04, 1.24]	0.99 [0.92, 1.08]	1.00 [0.93, 1.08]	1.04 [0.96, 1.13]

HR = Hazard ratio, ICD-10 = International Classification of Diseases, 10th revision, OR = Odds ratio, y = years.

[a] Information on the children's diagnoses were obtained from the database-linked Danish National Hospital Register, which contains information on all diagnoses of inpatients and outpatients in Danish hospitals. In each diagnostic category, we used the initial diagnosis.

[b] Data were split at indicated times.

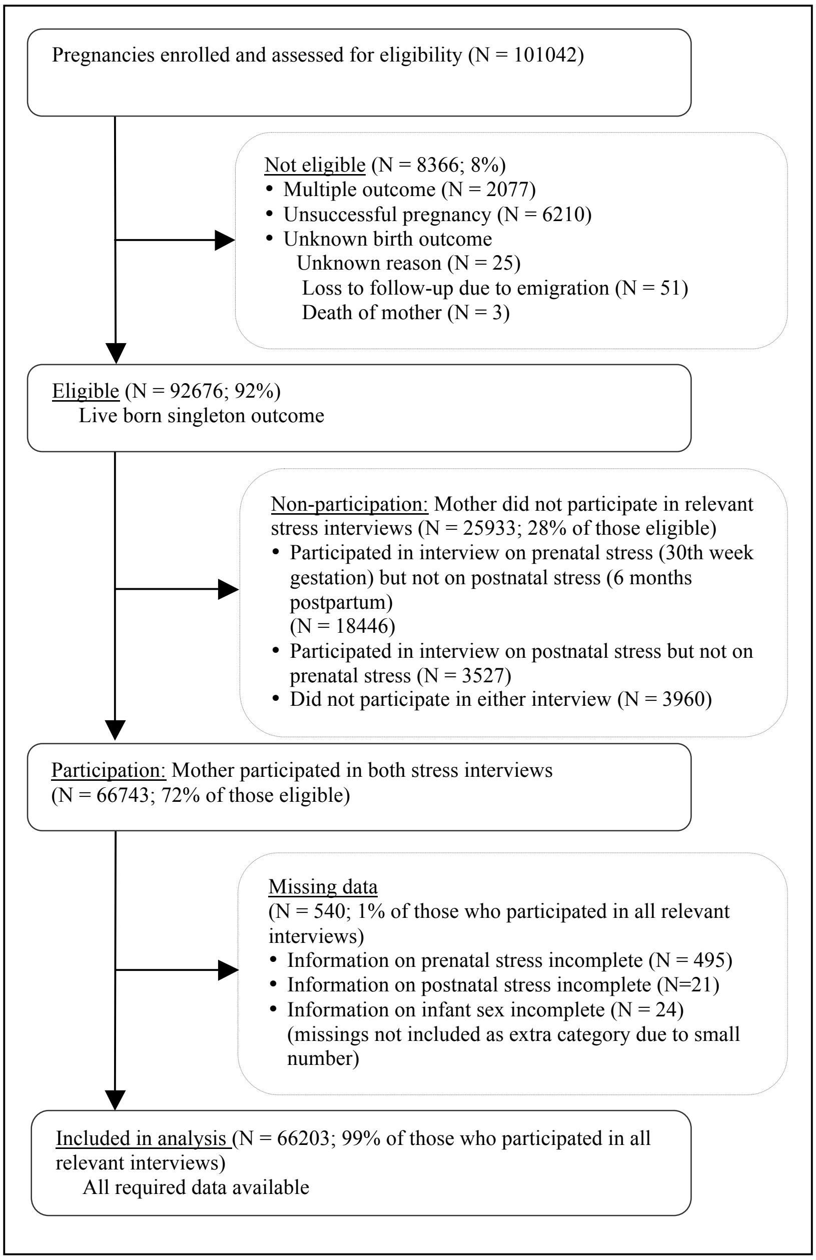

Supplemental Material Figure 1: Flowchart of Study Participants.

1a

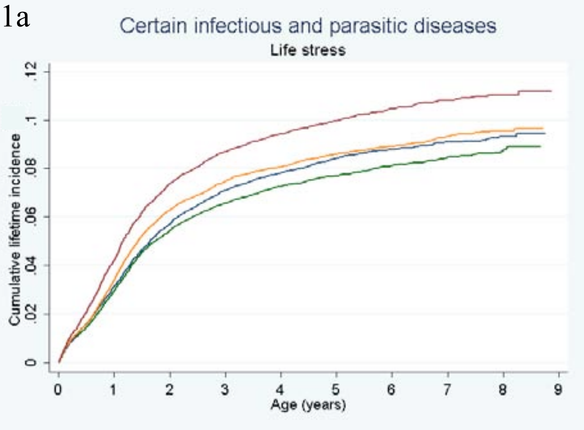

1b

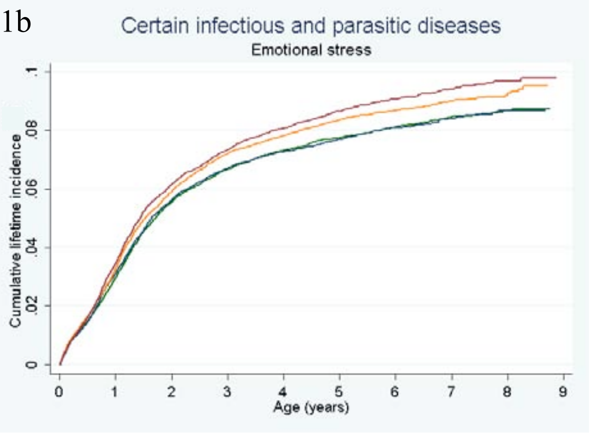

2a

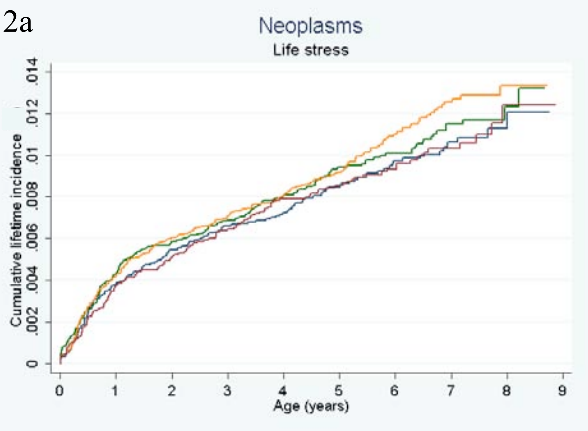

2b

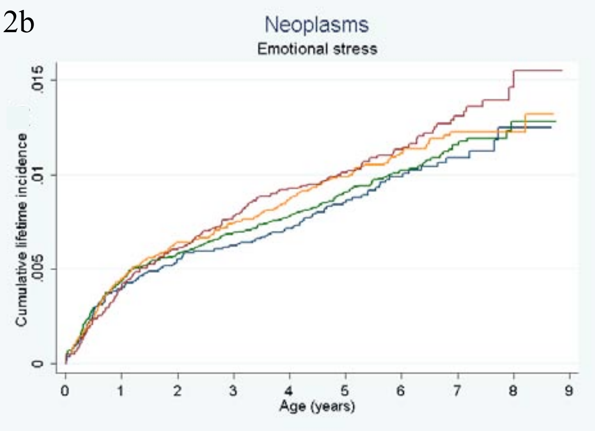

3a

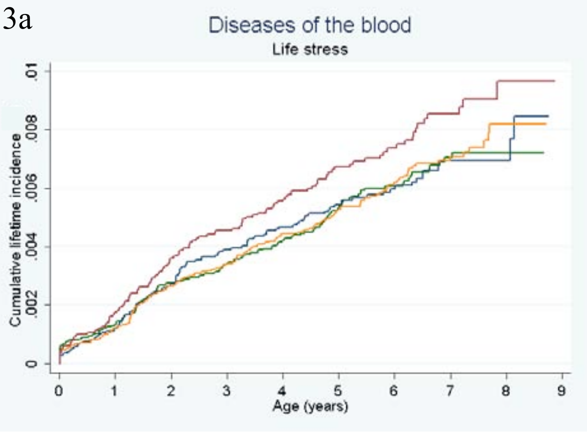

3b

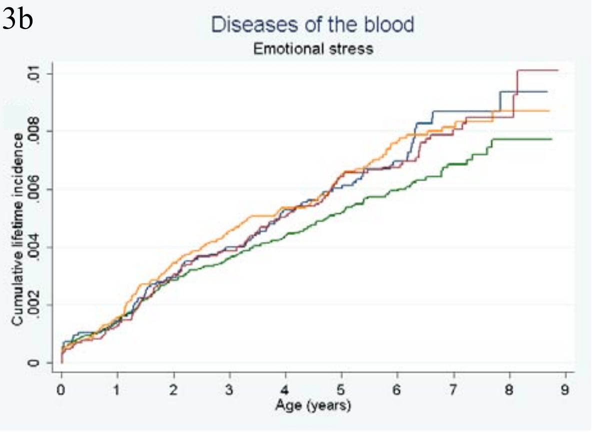

4a

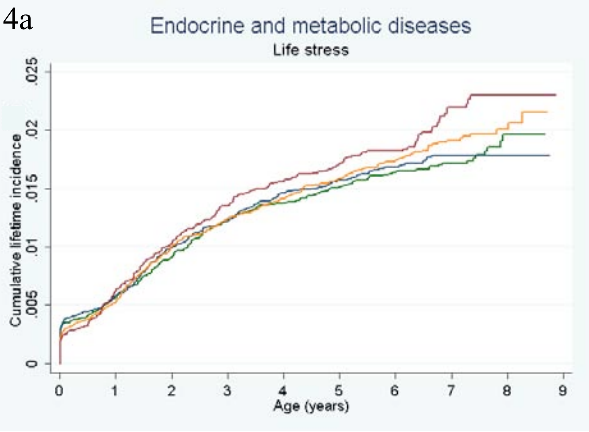

4b

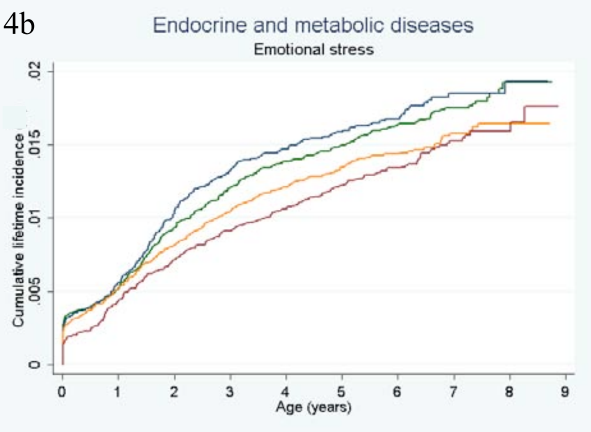

5a

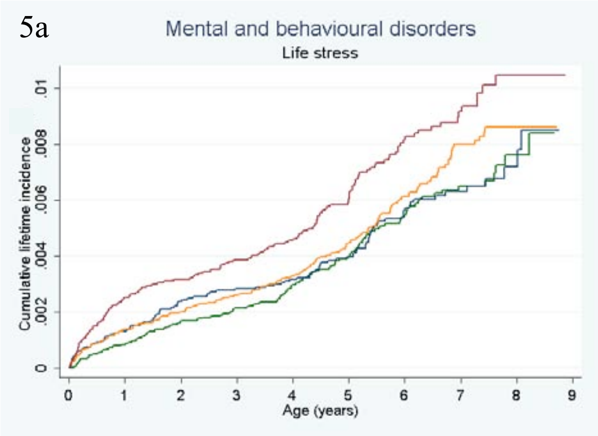

5b

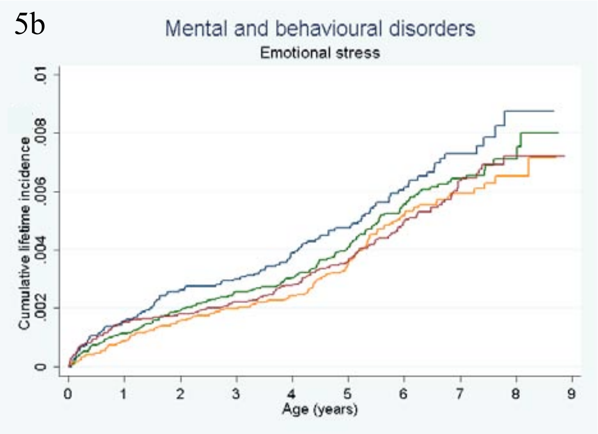

6a

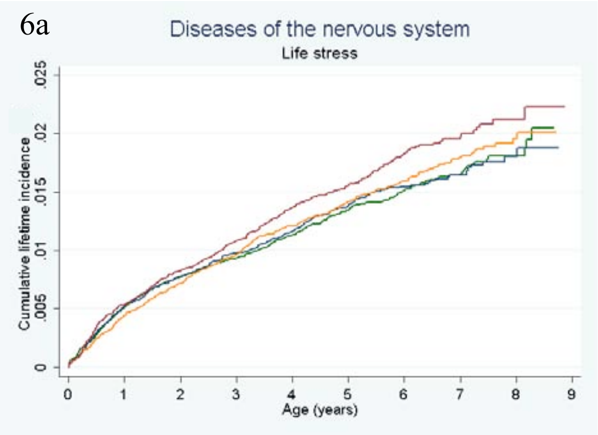

6b

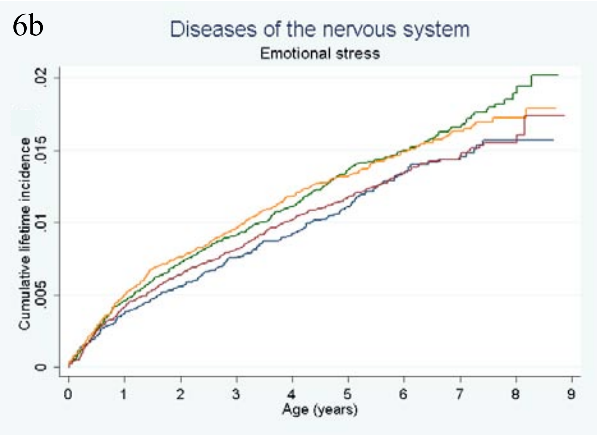

7a

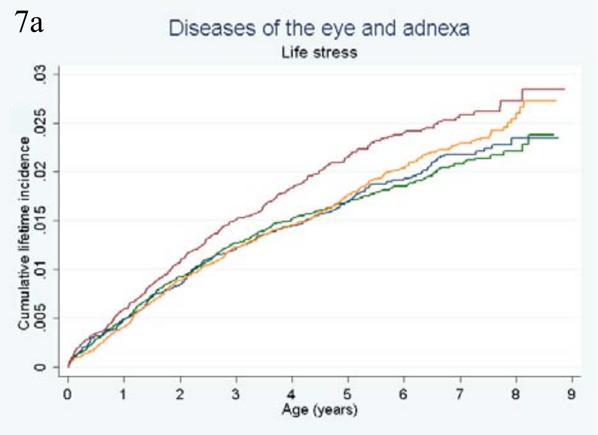

7b

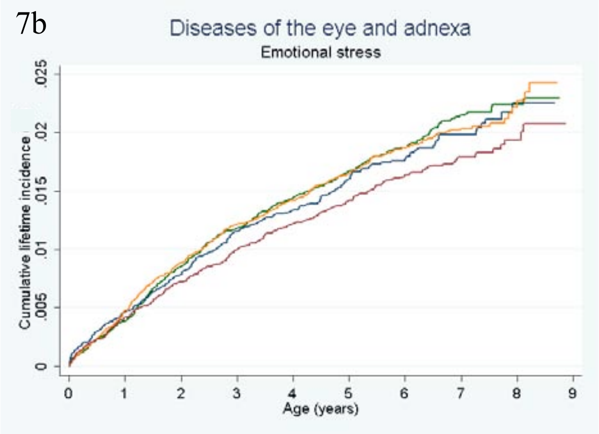

8a

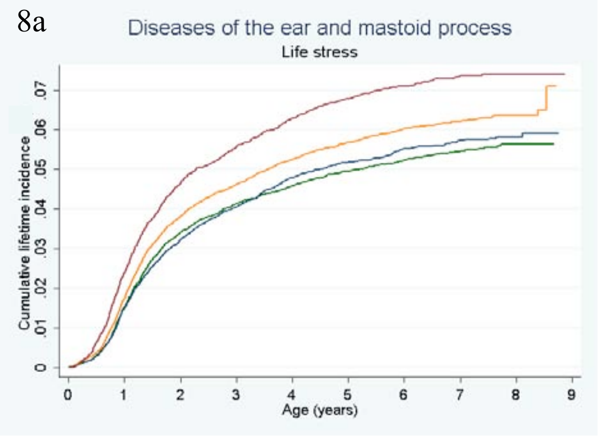

8b

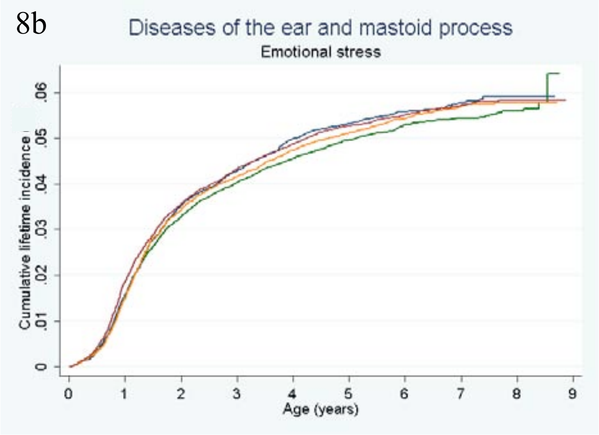

9a

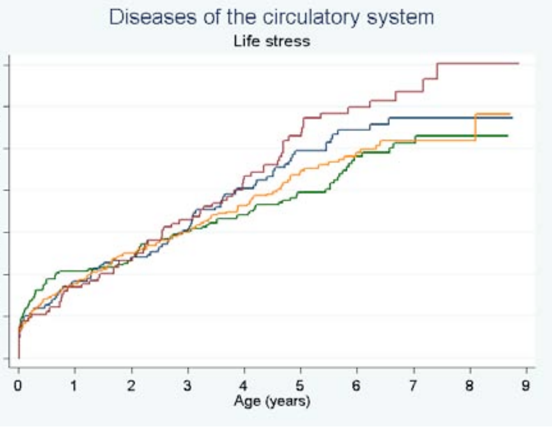

9b

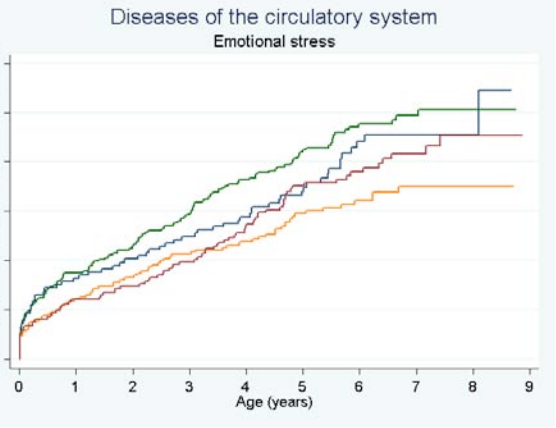

10a

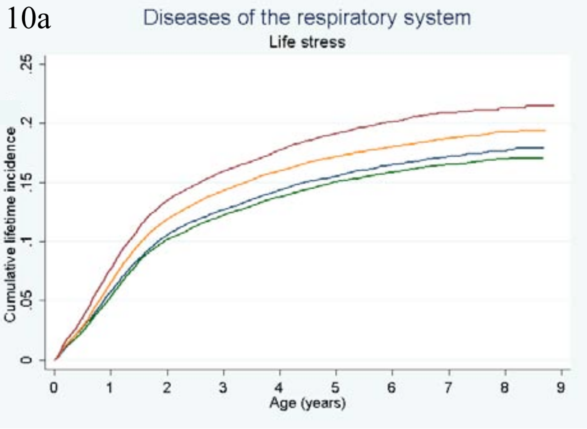

10b

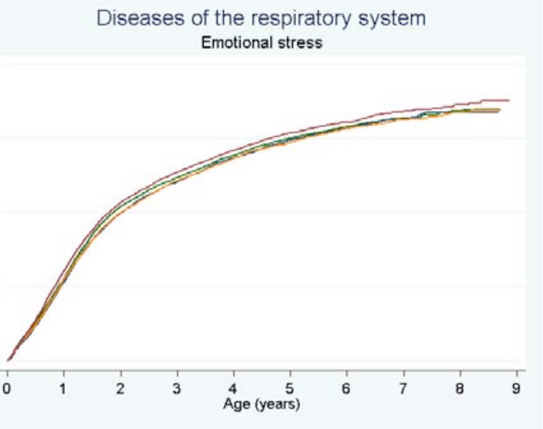

11a

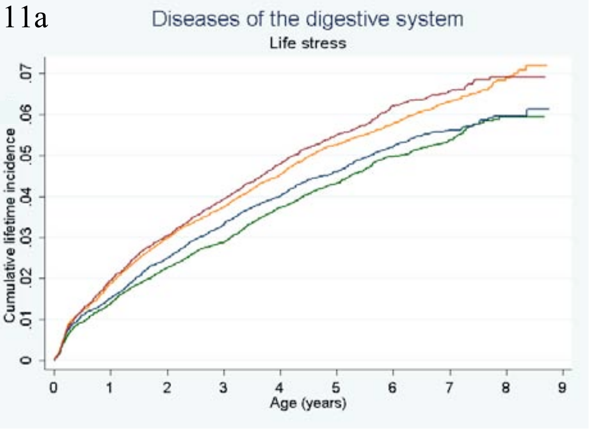

11b

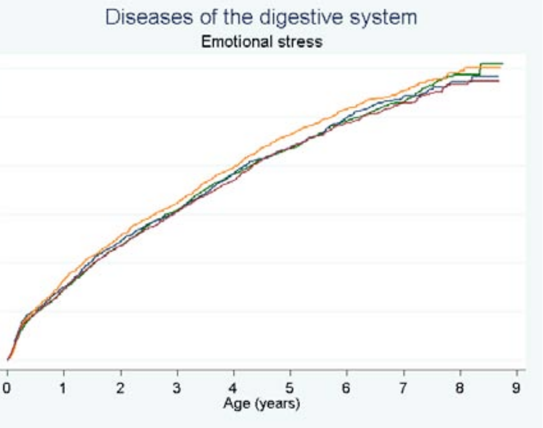

12a

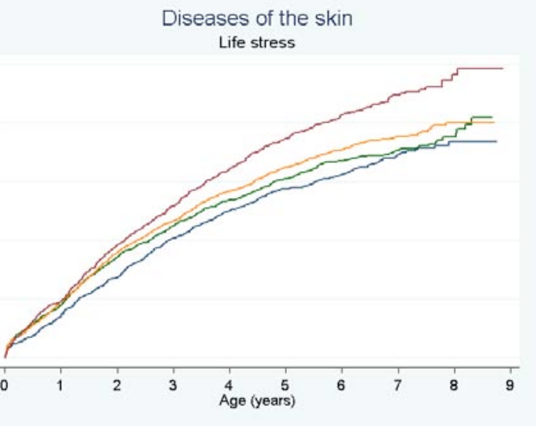

12b

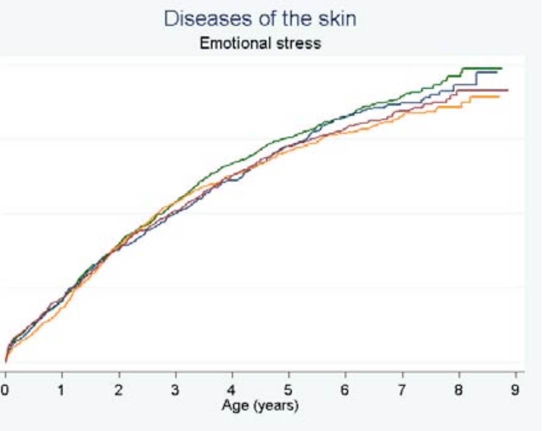

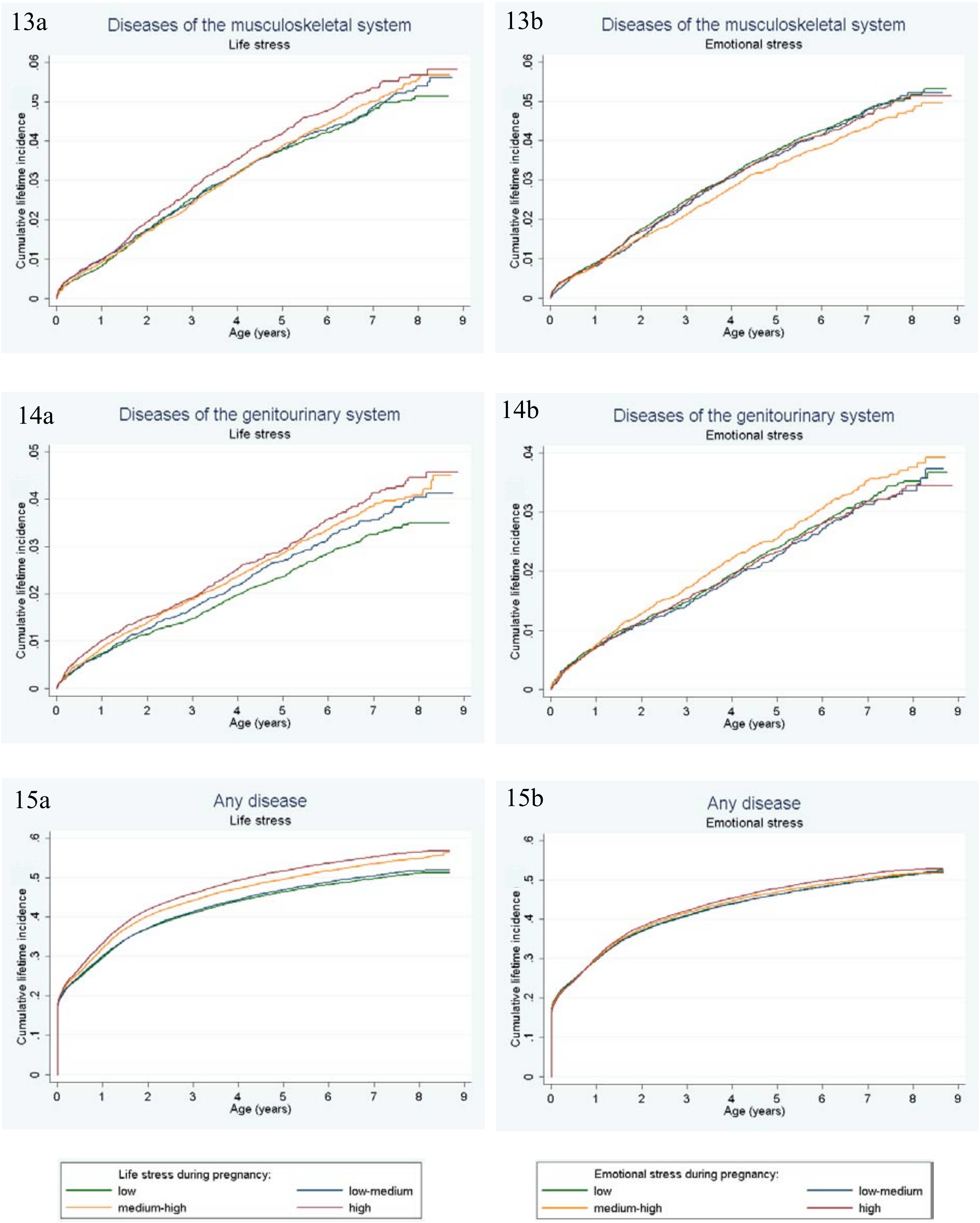

Supplemental Material Figure 2. Cummulative Lifetime Incidences of Offspring Disease by Stress During Pregnancy.
Cumulative lifetime incidence of offspring disease in diagnostic categories according to maternal psychosocial stress during pregnancy. Cumulative lifetime incidence of certain infectious and parasitic diseases (1a, b), neoplasms (2a, b), diseases of blood (3a, b), endocrine and metabolic diseases (4a, b), mental and behavioral disorders (5a, b), diseases of the nervous system (6a, b), diseases of the eye and adnexa (7a, b), diseases of the ear and mastoid process (8a, b), diseases of the circulatory system (9a, b), diseases of the respiratory system (10a, b), diseases of the digestive system (11a, b), diseases of the skin (12a, b), diseases of the muscoloskeletal system (13a, b), diseases of the genitourinary system (14a, b), and of any disease (15a, b) in the offspring, according to maternal life stress (left column, a) and emotional stress (right column, b) during pregnancy.

Supplemental Material References:

Bland JM, Altman DG. 2002. Statistics notes: Validating scales and indexes. BMJ 324:606-607.

Danish National Board of Health. 1993. Classification of Diseases. 10th Revision. Copenhagen.

Kanner AD, Coyne JC, Schaefer C, Lazarus RS. 1981. Comparison of two modes of stress measurement: Daily hassles and uplifts versus major life events. J Behav Med 4:1-39.
